# Clinical significance of exercise‐induced hypoalgesia in individuals with temporomandibular disorders and neck pain: A clinical trial protocol

**DOI:** 10.1113/EP091879

**Published:** 2025-02-24

**Authors:** Luiz Felipe Tavares, Ana Izabela Sobral de Oliveira‐Souza, Vladimir Aron, Ana Beatriz Oliveira, Henrik Bjarke Vaegter, Susan Armijo‐Olivo

**Affiliations:** ^1^ Postgraduate program in Physical Therapy (PPGFT) Federal University of São Carlos São Carlos Brazil; ^2^ University of Applied Sciences Osnabrück Faculty of Business Management and Social Sciences Osnabrück Germany; ^3^ Université Catholique de Louvain, Faculty of Medicine Institute of Neuroscience (IoNS) Brussels Belgium; ^4^ Pain Research Group, Pain Center Department of Anesthesiology and Intensive Care Medicine, University Hospital Odense, Department of Clinical Research, Faculty of Health Sciences, University of Southern Denmark Odense Denmark; ^5^ Faculties of Rehabilitation Medicine and Dentistry University of Alberta Edmonton Canada

**Keywords:** chronic pain, minimal clinically important difference, pain measurement, physical exercise

## Abstract

Evidence reports positive effects of neck motor control and aerobic exercises (AEs) to improve pain in individuals with temporomandibular disorders (TMD) and neck pain. A single bout of exercise typically leads to an increase in pain thresholds up to 30 min post‐exercise, known as exercise‐induced hypoalgesia (EIH). Studies evaluating the effects of aerobic and neck motor control exercises on EIH in individuals with chronic neck pain and TMD are limited. Measuring treatment effects and determining the clinical significance based on exercise types and loads and EIH response can improve clinical outcomes and adherence to exercise programmes. This study was designed to determine the clinical significance of EIH after neck motor control and aerobic training in participants with TMD and neck pain. Participants between 18 and 60 years with neck pain and/or TMD will be randomized to neck motor control or aerobic training groups. Participants will be assessed before, immediately after and 15 min after three treatment sessions within a 12‐week exercise programme. Assessments will include pain intensity, pressure pain thresholds and tolerance of masticatory and neck muscles, and the Global Rating of Change Scale. EIH response will be calculated in absolute and relative changes by subtracting the post‐ from the pre‐exercise values. Distribution‐based (e.g., effect size) and anchor‐based (e.g., receiver operating characteristics) methods will be performed to determine the clinical significance of EIH (minimal important difference).

## 1 INTRODUCTION

Musculoskeletal disorders are among the most costly health problems and are the leading causes of disability (Vos, 2012). One example is temporomandibular disorders (TMD), which are frequently associated with headaches and neck pain, and constitute a large part of public health expense (Vos et al., 2017). In addition, neck pain is the 11th leading cause of years lost to disability (Abbafati et al., 2020). Individual, ergonomic, sociocultural and psychosocial risk factors contribute to neck pain occurrence and chronicity (de Melo Castro Deligne et al., 2021). A high comorbidity between TMD and neck pain has been reported, mainly due to the close relationship among the neck, orofacial and cranial regions, with about 70% of patients with TMD reporting neck complaints (de Oliveira‐Souza et al., 2020; Silveira et al., 2015).

Neck muscle exercises are effective in reducing painful symptoms related to TMD (Armijo‐Olivo et al., 2016) and neck pain (Falla et al., 2013; Ris et al., 2016). Specific neck training using motor control exercises reduces the risk of neck pain recurrence and improves muscle strength, endurance, range of motion and neck motor control (De Pauw et al., 2019). Such effects are likely driven by physiological and biomechanical factors (Gross et al., 2015).

In addition, whole‐body exercises could reduce pain in patients with chronic pain conditions (Geneen et al., 2017). Previous studies reported that aerobic exercises (AEs) improve the quality of life and mental health of individuals with fibromyalgia, obesity, depression and headaches (Ambrose & Golightly, 2015; Ferro Moura Franco et al., 2021), and that a large number of individuals with TMD are sedentary or irregularly active (Tavares et al., 2024). Regular exercise, especially AE (i.e., an exercise subtype targeting large muscle groups and performed over an extended period of 20–60 min), promotes pain relief potentially by increasing serotonin and opioid levels in central pathways involved in the modulation of nociception (Lima et al., 2017; Rice et al., 2019; Stagg et al., 2011). However, limited evidence is reported on the effects of AEs on pain in individuals with both TMD and neck pain (de Oliveira‐Souza, Gülker, et al., 2024, 2024, de Oliveira‐Souza, Kempe et al. 2024, 2024). Therefore, a multimodal treatment including aerobic and strengthening exercises could improve the pain and function of individuals with craniocervical pain (Booth et al., 2017).

In pain‐free populations, a single session of exercise (e.g., aerobic, isometric, dynamic) typically leads to an increase in pain thresholds that can last up to 30 min, known as exercise‐induced hypoalgesia (EIH) (McPhee Christensen et al., 2022; Moana‐Filho et al., 2018; Nasri‐Heir et al., 2019; Wewege & Jones, 2021). Several mechanisms may contribute to EIH, including the activation of the opioid and cannabinoid systems, release of hormones and neurotransmitters (e.g., endorphins, enkephalins) in response to induced stress, changes in the cardiovascular system, and pain modulation by the central nervous system (De la Corte‐Rodriguez et al., 2024; Vaegter & Jones, 2020). A single session of isometric, submaximal aerobic, and range of motion exercises reduces local (painful area) and remote (non‐painful area) pain sensitivity in individuals with chronic neck pain (Senarath et al., 2023). However, submaximal AEs have only been evaluated in chronic whiplash disorders, and the meta‐analysis included only observational studies (Senarath et al., 2023), meaning that clinical trials evaluating the effects of aerobic and specific exercises on EIH in individuals with chronic neck and TMD pain are limited, with low quality of evidence and uncertain risk of bias (Wewege & Jones, 2021). Another review stated that despite several studies being conducted on EIH in different chronic conditions, only a minority used randomized controlled trial designs (Vaegter & Jones, 2020).

Moreover, identifying which exercise parameters yield the strongest hypoalgesic effects can help clinicians to choose the best intervention approaches. In this context, the concept of clinical significance is highly important to understand and interpret the results of clinical research. Clinical relevance or significance is roughly defined as the value or importance of the effect of an intervention based on the patient's perspective (Armijo‐Olivo et al., 2022). In this scenario, the patient's perception regarding changes after treatment can be more meaningful than the statistical result (i.e., a statistically significant result does not guarantee clinical improvement). Thus, measuring the clinical significance of an intervention and tailoring the intervention based on EIH response can benefit clinical settings, and improve adherence to exercise programmes, which is a challenge in chronic pain populations (Ambrose & Golightly, 2015). However, the reporting of clinical significance in rehabilitation studies for individuals with chronic pain is still insufficient (Armijo‐Olivo et al., 2022). Furthermore, the need to establish the minimal important difference (MID) of EIH, to ensure blinding of assessors, and to consider patients expectations regarding the treatment when evaluating EIH has been highlighted in the literature (Aron et al., 2024).

In this context, an underestimation of the acute effects of physical activity on managing chronic pain has been reported (Schofield et al., 2011). Thus, it is important to explore how a single bout of exercise affects pain and the clinical significance of EIH in individuals with neck pain and TMD. Therefore, this study was designed to fill in this gap.

## 2 OBJECTIVES

To evaluate whether a single session of neck motor control exercises and aerobic training can cause EIH by measuring pain intensity, pressure pain thresholds (PPT) and tolerance (PPTo) in individuals with TMD and neck pain.

To evaluate EIH response at different time points during a 12‐week progressive neck motor control exercise and aerobic training treatments.

To determine the clinical significance of EIH, by calculating the MID of it based on distribution and anchor‐based methods.

## 3 METHODS

### 3.1 Study design

**FIGURE 1:**
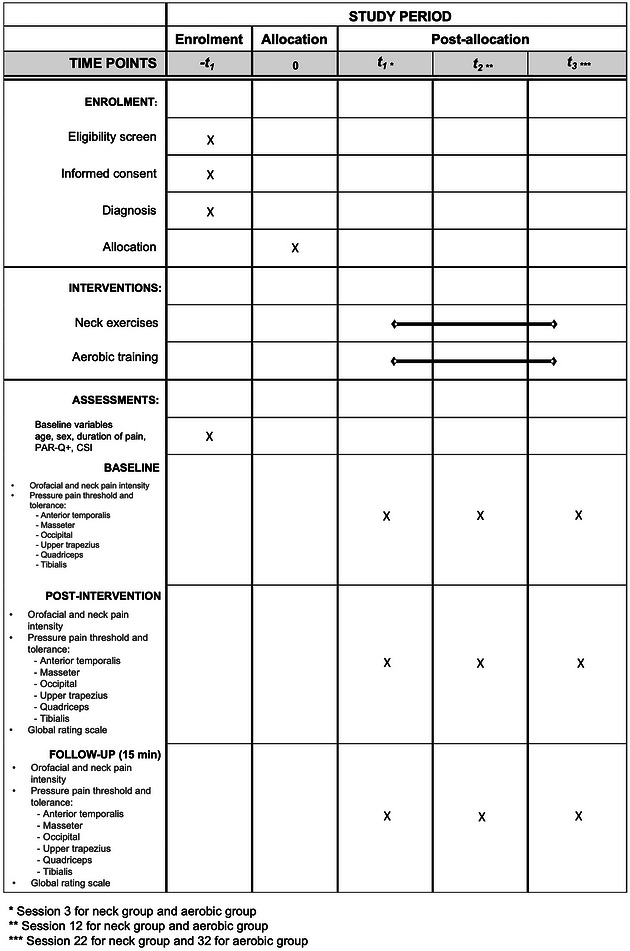
Schedule of enrolment, interventions and assessments. CSI, central sensitization inventory; PAR‐Q+, Physical Activity Readiness Questionnaire.

This study will be a parallel pilot randomized controlled clinical trial conducted at the University of Applied Sciences, Osnabrück, Germany. This study will be part of an umbrella project and will include participants recruited from a larger study previously registered in clinicaltrials.gov (NCT05232604). This project has also been approved by the human research ethics committee of the University of Applied Sciences (HSOS/2020/2/1) and will be conducted in accordance with the ethical principles of the *Declaration of Helsinki*. Figure 1 details the overall study period of enrolment, assessments and interventions. This protocol follows the guidance of the Standard Protocol Items: Recommendations for Interventional Trials (SPIRIT) statement.

### 3.2 Participants

Women between 18 and 60 years of age, diagnosed with chronic non‐specific neck pain (as described by the IASP) and/or diagnosed with TMD according to the Diagnostic Criteria for Temporomandibular Disorders—DC/TMD (Dworkin, 2014) will be included. In addition, a baseline pain score of at least 30 points on a 0 to 100 visual analogue scale (VAS) will be required. Participants with severe pathologies (red flags) related to neck pain, reporting comorbidities (e.g., fibromyalgia), diagnosed with psychiatric disorders, and/or who received therapy within 3 months before study entry will be excluded. All participants will complete the Physical Activity Readiness Questionnaire (PAR‐Q+) to assess readiness to exercise. The Central Sensitization Inventory (CSI) recommended for patients with chronic pain will also be applied. The CSI is an easy‐to‐apply self‐report questionnaire that tracks and assesses symptoms related to overall sensory disturbances (Mayer et al., 2012).

This exploratory clinical trial aims to gather preliminary data on the safety and potential efficacy of a single bout of exercise for individuals with TMD and neck pain. Given the exploratory nature of this study, a formal sample size calculation was not conducted. Instead, the sample size was determined based on practical and logistical considerations. This exploratory study pilot will initially target 30 participants (15 per group), aiming to gain insights and assess feasibility. Based on the targeted sample size and a clinically important difference of 19 points (on a 0–100 pain scale) (Calixtre et al., 2020) with a standard deviation of 20, the study has an estimated 71% power to detect a large effect size (ES, Cohen's *d* = 0.95) using a significance level of 0.05. The recruitment rate of participants, availability of the research team, time, and resources were considered for the targeted sample size. The data collection is expected to be finished by the end of 2025. The results of this study will help to design and calculate sample sizes for future studies.

### 3.3 Allocation concealment

All participants will be randomly assigned to one of the treatment groups: neck motor control exercises or AEs by using an automated system hosted on a web platform. The randomization sequence will be stratified by chief complaint (neck pain or jaw pain) and age (18–30, 31–45, 46–60) and generated by an external party using a randomization website. The sequence generated will be automatically programmed in REDcap to allow the allocation concealment of the sequence.

### 3.4 Treatment interventions

#### 3.4.1 Aerobic exercise (AE)

AE training will consist of 12 weeks of exercise supervised by a physiotherapist with a frequency of three times per week (36 sessions in total). Overall, the session will have a total duration of 60 min and will be divided into three parts: warm‐up, main exercise period and cool‐down. The training will be performed on a cycle ergometer or treadmill (based on the participant's choice). The intensity will be based on maximum heart rate (HR_max_), heart rate reserve (HR_res_) and the subjective perception of effort (Borg Scale), which will be monitored. The Borg scale will be the main variable considered to increase the intensity of the protocol or to stop the training. The HR_max_ will be calculated by a ramp protocol for the cycle ergometer and confirmed by standard formulas.

The intensity of the AE programme will be progressively increased according to each participant's response but will be standardized as much as possible, as follows:

1. In the first 2 weeks, participants will start the walking/cycling programme at low intensity (at 60% HR_max_ or 9–11 on the Borg scale).
2. At 2–6 weeks, depending on the response to the first sessions, the training will progress to moderate intensity (55–70% HR_max_ or 12–14 on the Borg scale);
3. In the last 6 weeks, high‐intensity interval training (HIIT) will be targeted as recommended by the literature (Geneen et al., 2017). The HIIT consists of changing between moderate and high intensity for short intervals. Thus, during the first 4 min, the participant should train at high intensity (75–90% HR_max_ or 15–17 on the Borg scale), and in the subsequent 3 min, the participant should train at moderate intensity (55–70% HR_max_ or 12–14 on the Borg scale). This is done continuously until the total training time is completed (4 min × 4 times (high‐intensity training) followed by 3 min of moderate in between, totalling 28 min of active training) according to the participant's response. The intensity of the aerobic training in each session will be targeted individually with an increase of 5 W every 5 min according to the specific stage the participant is in and based on the Borg scale. Supplementary Material 1 describes the full protocol for the aerobic training group, which will also perform stretching exercises before and after the intervention.

#### 3.4.2 Neck motor control exercises

The duration will consist of 12 weeks with a frequency of three times per week in the first month, two times in the second month, and once per week in the final month (24 sessions in total). Each session will include localized motor control of neck flexors and extension muscles with a duration of 30–45 min. Low‐load craniocervical exercises (head nods) will be performed in the early stages (first 6 weeks) guided by visual feedback from a pressure unit (NOD device). Higher load neck exercises will be performed in later stages (last 6 weeks). This duration of treatment is commonly used in clinical settings and has been shown to be sufficient to improve clinical outcomes (Bednarczyk et al., 2024). This exercise protocol is detailed in Supplementary Material 2 and has been successfully tested in subjects with neck pain.

### 3.5 Assessments

#### 3.5.1 EIH procedures and time points

On the days of assessments, participants will be instructed to refrain from additional physical exercise, caffeine, nicotine and alcohol (Vaegter et al., 2019). To evaluate the acute effects of exercise protocols, participants will be assessed before, immediately after and 15 min after a single intervention session. The sessions are part of a 12‐week exercise programme. Since both protocols progressed from light to high intensity throughout the intervention period, three time points (i.e., three sessions) were chosen for the evaluations of three different exercise intensities (low, moderate and high). For the neck motor control exercises group, sessions 3, 12 and 22 were chosen. For the AE group, sessions 3, 12 and 32. Assessors will be blinded to the treatment allocation. Those specific sessions were chosen to ensure that participants will be assessed at the beginning, middle and end of each specific protocol at low, moderate, and high intensities, respectively.

**FIGURE 2:**
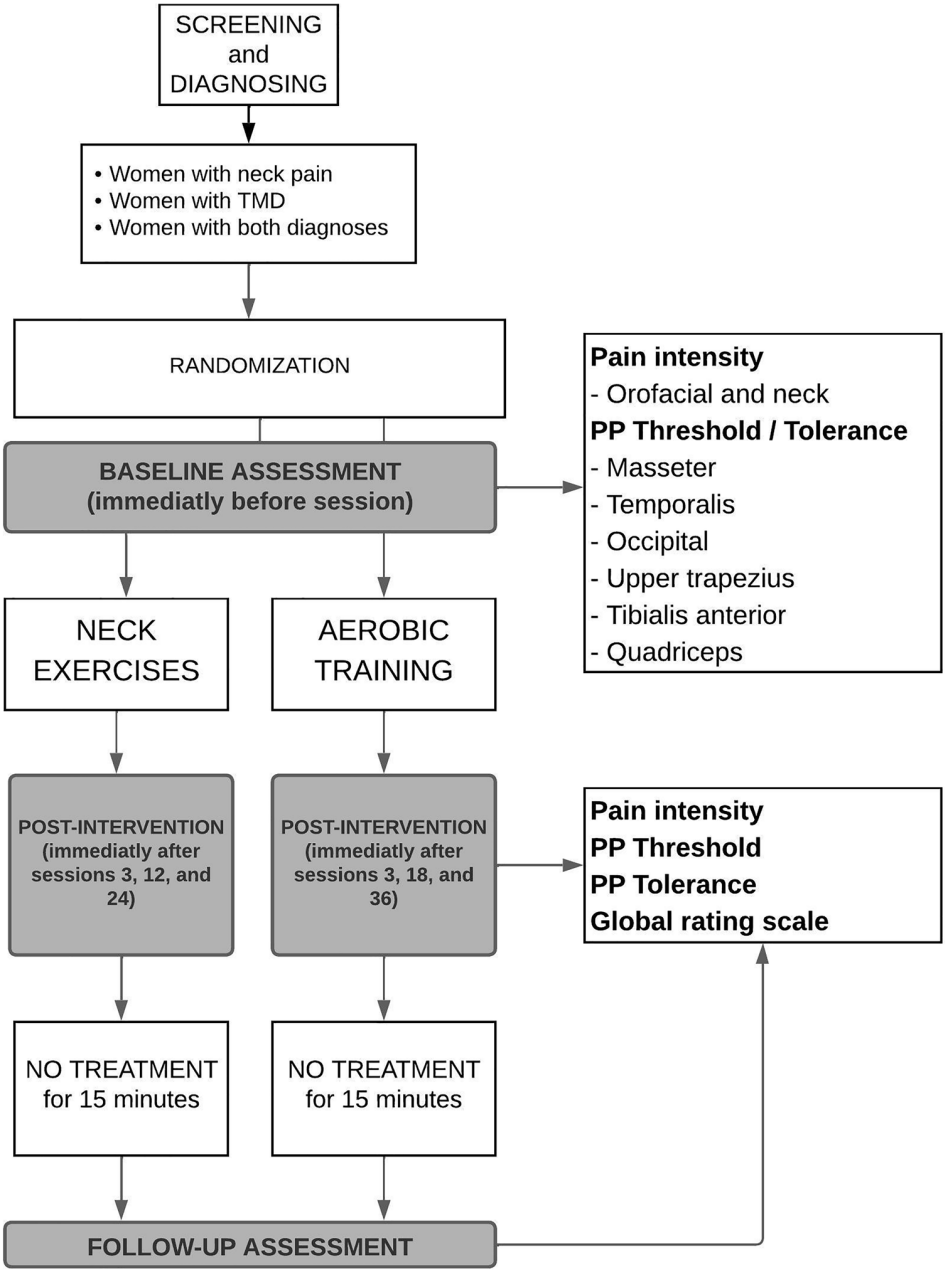
Flowchart of study procedures.

At the three time points, three measurements will be conducted. The first measurement will be collected before the intervention, the second measurement will be immediately after the end of the intervention (considering 5 min of preparation of the room) and the third measurement will be after 15 min of rest on a stretcher (30 min after intervention). During the resting time, no intervention will be performed. Considering that each measurement lasts approximately 15 min, this timeline will allow us to determine whether EIH lasted at least 30–35 min. Figure 2 describes the sequence of events.

### 3.6 Outcome measures

#### 3.6.1 Pain intensity

Pain intensity will be measured by the VAS in which the participants will rate the pain from 0 to 100 mm, referring to the orofacial region (TMD) and the cervical region, separately, considering that 100 indicates the ‘worst imaginable pain’ and 0 indicates ‘no pain’. The VAS has good reliability (Jensen et al., 1999), and a 19 mm reduction has been considered clinically important for women with TMD (Calixtre et al., 2020).

#### 3.6.2 Quantitative sensory testing

##### PPT

**FIGURE 3:**
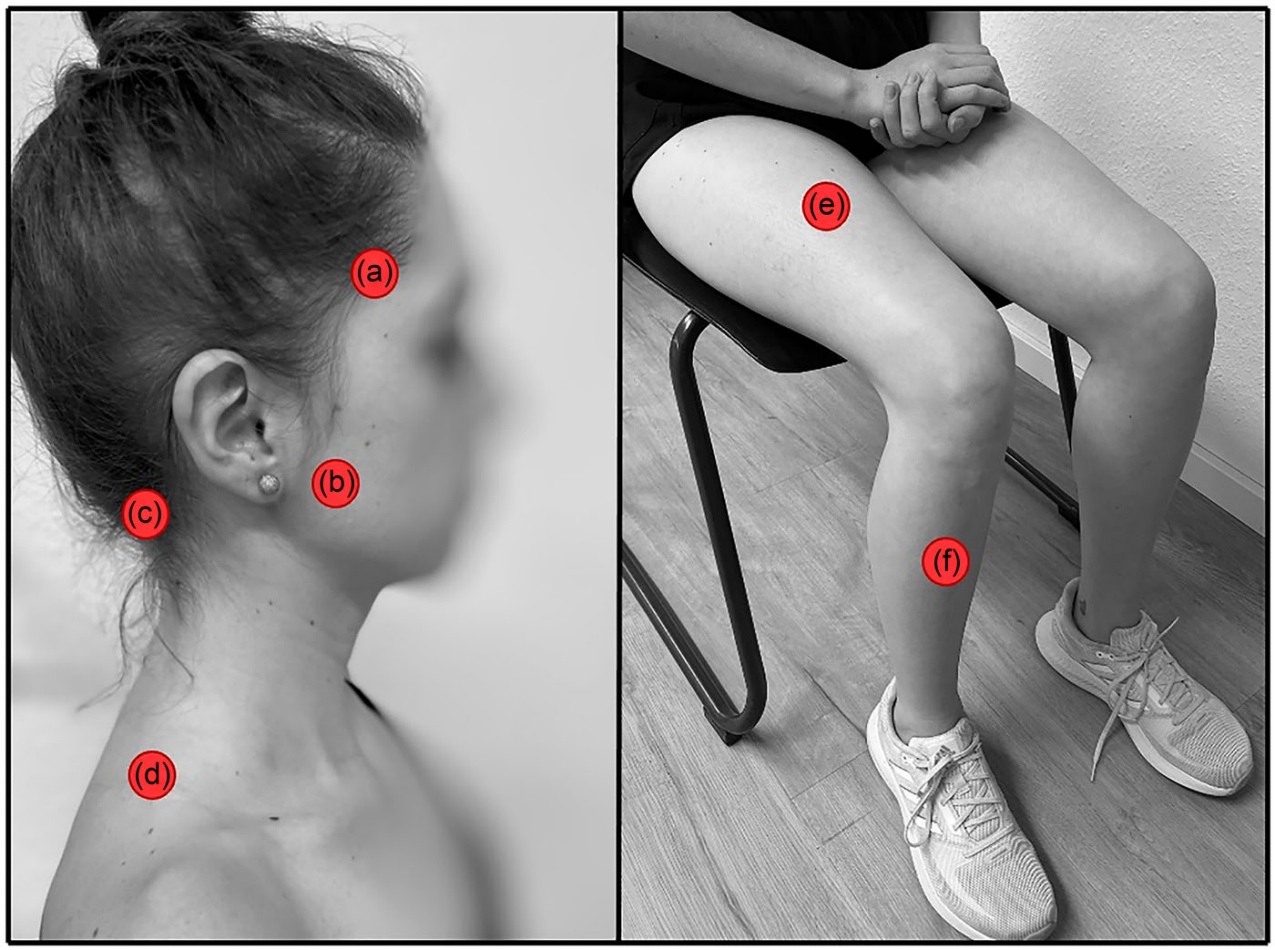
Masticatory, neck, and leg muscles chosen for assessments. (a) anterior temporalis; (b) superficial masseter; (c) occipital region; (d) upper trapezius; (e) quadriceps; (f) tibialis anterior.

The PPT is defined as the minimum pressure that induces the first sensation of pain. PPT will first be assessed on the dominant hand for familiarization. Specific muscles were selected because they are the most prone to pain in this population and will be assessed bilaterally (Figure 3) (Silveira et al., 2015):

1. Anterior temporalis (2 cm above the zygomatic arch).
2. Superficial masseter (1 cm superior and 2 cm anterior to the mandibular angle).
3. Occipital region (slightly below the superior nuchal line where the trapezius muscle inserts).
4. Upper trapezius (halfway between the C7 vertebra and the acromion).

Additionally, one measurement will also be taken in two muscles located in the dominant lower extremity:

1. Quadriceps (rectus femoris, 20 cm proximal to the base of patella) (Jones et al., 2019; Vaegter et al., 2015).
2. Tibialis anterior (muscle belly) (Alburquerque‐Sendín et al., 2013; Ashina et al., 2006; Walton et al., 2011)

**FIGURE 4:**
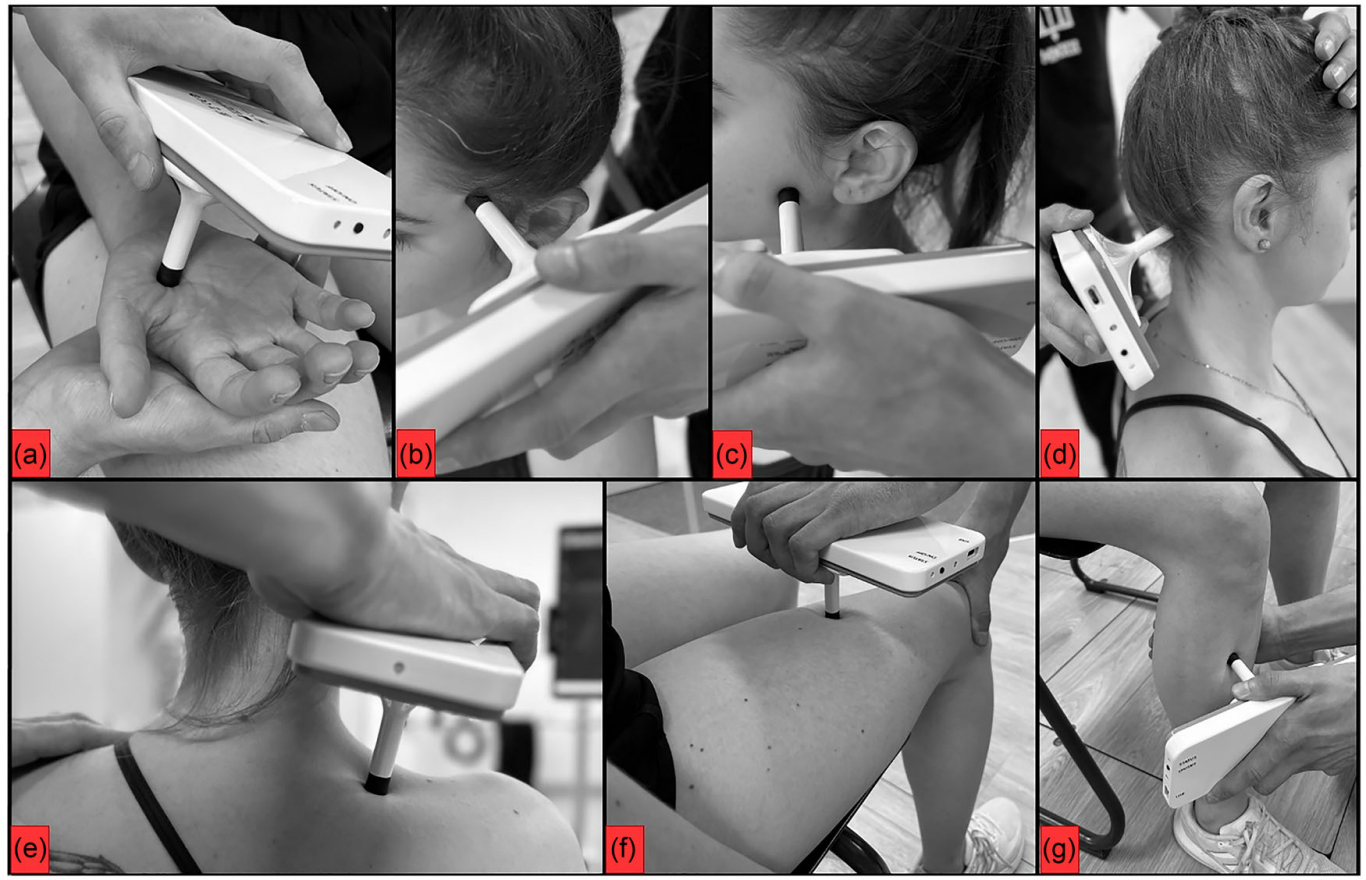
NOD device assessment in the dominant hand (a), left anterior temporalis (b), left masseter (c), right occiput (d), right upper trapezius (e), dominant quadriceps (f), and dominant tibialis anterior (g).

The algometer function of the NOD device (http://www.to‐nod.com/) will be used to measure PPTs and pressure pain tolerance (PPTo). The measurements will be collected in kPa and converted to kg/cm^2^. The order of locations to be tested will be randomized, and a rate of 0.5 kg/s will be used as force application (Figure 4). The mean of two PPT measurements will be considered for analysis. If the second measure differs more than 10% from the first measure, a third measure will be collected. The minimal detectable change for the PPTs of neck muscles of patients with neck pain was reported as 0.45–1.13 kg/cm^2^ and for women with TMD as 0.18 kg/cm^2^ and 0.22 kg/cm^2^ in the masseters and temporalis (Calixtre et al., 2020), respectively. The PPT measurements have good to excellent inter‐ and intra‐rater reliability (0.74–0.99) (Walton et al., 2011). Higher values mean less pain sensitivity.

#### 3.6.3 Pressure pain tolerance

The pressure pain tolerance (PPTo) will be considered as the most bearable pain. Just one measurement will be collected for PPTo in each location, and this measurement will be conducted immediately after the two measurements of the PPT.

#### 3.6.4 EIH

For participants with neck pain, the occipital region point will be chosen to calculate EIH, and for TMD, the masseter point will be used. Relative and absolute changes will be used to calculate EIH. The pre‐exercise PPT values will be subtracted from the post‐exercise values (Aron et al., 2024; McPhee Christensen et al., 2022). After subtracting the values, an increase in the thresholds will be considered a positive response to exercise. The difference between the pre‐ and post‐exercise values of the VASs will also be assessed.

##### Self‐perception of improvement

As an anchor measure, the Global Rating of Change Scale (GRCS) will be collected along with pain thresholds at each time point for each group (Kamper et al., 2009). This tool was designed to quantify a patient's improvement or deterioration over time, being important to determine the effect of the intervention based on the patient's perspective. Participants will classify post‐intervention effects on a 15‐point scale from −7 (a very great deal worse) to 7 (a very great deal better). The GRCS will be used to determine the clinical significance of the EIH.

### 3.7 Analyses plan

All analyses will be performed by the statistical program SPSS version 26 (IBM Corp., Armonk, NY, USA) and STATA v.17 (StataCorp, College Station, TX, USA). The Shapiro–Wilk test and visual inspection of histograms and graphs will be used to verify the normality of the data. Then, descriptive statistics will be performed to characterize the sample and explore variables. The results will be presented as mean and standard deviation when the variables are normally distributed, or as median and interquartile range when the data distribution is not normal. A 95% confidence interval will be adopted. *P*‐values will be interpreted based on Sterne and Smith to determine the evidence against the null hypothesis (Sterne & Smith, 2001).

To accomplish objective (1), which is to assess the effects of a single bout of neck motor control exercises and aerobic training exercises on pain intensity, PPT and PPTo in participants with TMD and neck pain, further inferential analyses will include separate mixed‐effects models (random effect for repeated measurements per participant) for each outcome to assess the effect of the intervention (groups) over time and adjusted by other covariates. First, a fixed‐effects model for each outcome at the final evaluation that includes only the intervention groups and baseline score will be created. Then, mixed‐effects models (random effects for repeated measurements per participant and groups (intercept and slope) will be tested. Finally, these models will be expanded to include covariates (e.g., demographics, psychosocial variables). Models will be compared using the Akaike information criterion (AIC) and the Bayesian information criterion (BIC). The covariates may be dropped from the models if not required for model fit based on AIC and BIC. Model estimates and associated 95% confidence intervals (CI) will be reported.

To accomplish objective (2), the EIH response will be calculated in absolute (raw score) and relative (percentage) changes by subtracting the post‐exercise PPT and PPTo values from the pre‐exercise values. Further exploratory analyses will also be performed with mixed‐effects models considering EIH as an outcome to compare the time points of treatment (beginner, intermediate and advanced training) and group treatment.

To determine the clinical significance of EIH (objective (3)), distribution‐based and anchor‐based methods will be performed after the two modalities of exercise. Using distribution based‐methods, ES will be calculated and will be classified following Cohen's *d* formula and interpretation (large ES if >0.8; moderate, >0.5; small >0.2) (Cohen, 1988), if the data are parametric, and by Cliff's Delta, if the data are non‐parametric (Macbeth et al., 2011). Other distribution‐based methods such as those derived from standard deviation and ESs will be explored (Armijo‐Olivo et al., 2022).

The MID of the outcomes of interest (PPTs and PPTo) for EIH using the GRCS as anchor will be used (anchor‐based methods). Participants will be classified as having no improvement when their responses to the GRCS are between −7 and 0, slight improvement between 1 and 3, moderate improvement between 4 and 5, and large improvement between 6 and 7 after the treatment. Changes from baseline to after intervention and 15 min will be calculated for each one of the outcomes of interest at each of the time points collected and used for analysis. Correlations between the GRCS and each of the variables of interest will be tested. The receiver operating curve analysis will be performed to generate the MIDs. This analysis compares the values of each measure from both groups according to the GRCS: participants who did and did not show minimal improvement after treatment. This method constructs a graph of the measures’ performance in classifying the groups for each possible cut‐off point and calculates the sensitivity and specificity for all the cut‐offs. The main analysis will concentrate on calculating the minimal improvement to calculate the MIDs and differentiate the groups. Secondary analysis will include moderate and large improvement when possible. In addition, and if possible, an analysis divided by motor areas (primary and non‐primary) for each group (neck or aerobic) and each diagnosis (TMD, neck only, and both) will be addressed.

## AUTHOR CONTRIBUTIONS

All authors have approved the final version of the manuscript and agree to be accountable for all aspects of the work in ensuring that questions related to the accuracy or integrity of any part of the work are appropriately investigated and resolved. All persons designated as authors qualify for authorship, and all those who qualify for authorship are listed.

## CONFLICT OF INTEREST

None declared.

## FUNDING INFORMATION

None.

## Supporting information



Supplementary material 1. Description of the exercise for the aerobic training group.

Supplementary material 2. Description of the neck exercises for the neck training group.
