# Supplementary material for: Clinical significance of exercise‐induced hypoalgesia in individuals with temporomandibular disorders and neck pain: A clinical trial protocol
Source: Exp Physiol. 2025 Feb 24;111(6):2777–86. doi: 10.1113/EP091879 (PMC13238672; doi:10.1113/EP091879)
Supplement: Supplementary file 1 — Supplementary material 1. Description of the exercise for the aerobic training group. [file EPH-111-2777-s002.docx]

Supplementary Material 1 – Description of the exercise for the aerobic training group.

**Equipment needed:**

- Blood pressure monitor
- Polar heart rate monitor
- Oximeter
- Borg scale
- Tablet and stand
- Cycle ergometer or Treadmill

**Warm-up**

First, the participants stretch to warm up (2 x 30 seconds per side, if possible; otherwise, 2 x 15 seconds per side). The stretching is done by the participants independently and while standing.

| **Stretching the neck extensors:**  Ask the participant to fold the hands and place them under the occiput in the neck. The thumbs rest on the lower part of the skull. Then, the participant bends the head in flexion and reinforces the stretch by pulling the arms slightly downwards. Make sure that the participant maintains an upright posture. | 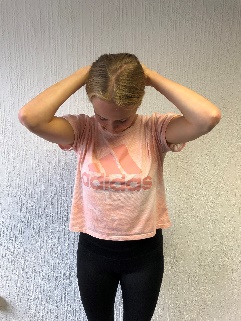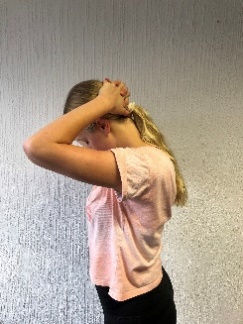 |
| --- | --- |
| **Stretching the lateral neck muscles**:  Ask the participant to bend their head sideways into lateral flexion. He then places their hand on the side of the head to increase the stretch. This should be felt in the middle area of the neck. | **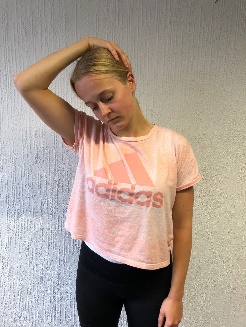** |
| **Levator stretch:**  Ask the participant to rotate the head in one direction and then bend it slightly. The participant reinforces the stretch by placing their hand on the side of the back of the head and pulling accordingly. A stretch should be felt in the dorsal part of the neck. | 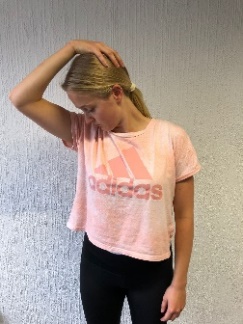 |
| **Stretching the posterior shoulder muscles**:  Ask the participant to hold one arm sideways in front of the body. It should be at about chest level. Then, take the other hand to the shoulder to be stretched and increase the pressure backwards. It is important that the participant does not pull their shoulder up. You should then feel a stretching sensation in the back of the shoulder. | 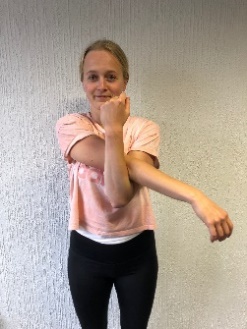 |
| **Stretching the triceps:**  Ask the participant to place one hand towards the back of the equilateral shoulder. The other arm reinforces the stretch by pulling the elbow backwards with the other hand. A stretching sensation should be felt in the course of the triceps and the axilla. During the execution, make sure that the participant does not move out into an increased hollow back. | 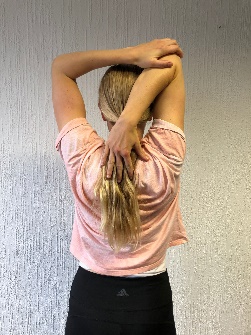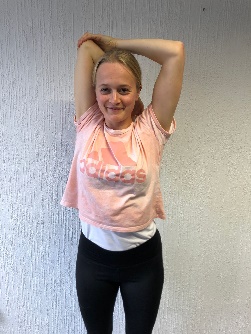 |
| **Stretching the pectoralis:**  The participant raises their arms sideways to 90° and bends the elbows also to 90°. He then stands in a door frame and places the palms of their hands on both sides of the wall. He stands in a crotch position and shifts their weight to the front foot so that both sides in the chest area are stretched equally. | 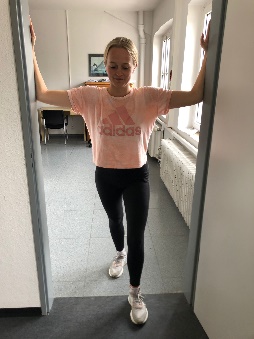 |
| **Stretching the quadriceps:**  Ask the participant to stand on one leg and bend the other leg so that the foot comes as far as possible towards the buttocks. It is important that the participant does not move into an increased hollow back and that the upper body remains upright. If there are problems with balance, the participant can hold on to an object or wall with one hand. The participant should feel a stretching sensation in the groin and front of the thigh. | 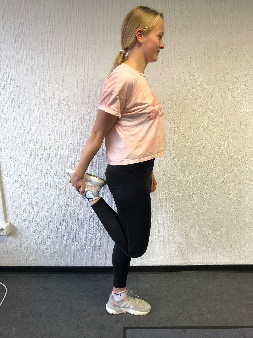 |
| **Stretching the adductors:**  Ask the participant to do a lateral lunge and shift their weight to one side. The upper body should remain as upright as possible, and the pelvis should not turn up sideways. A stretching sensation should only be felt on the inside of the leg. | 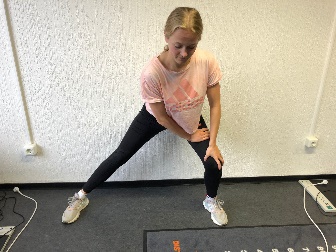 |
| **Stretching the posterior chain:**  Ask the participant to stand hip-width apart and bend forward. The legs should be stretched as much as possible, and the hands should move as far as possible towards the floor. A stretching sensation should be felt all over the back of the body, but especially on the back of the legs. | 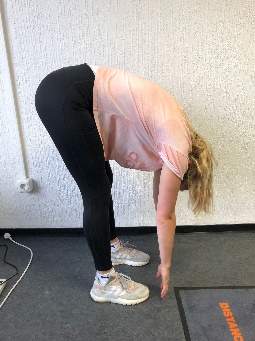 |

After the stretches, the saddle height is adjusted to about hip height, and the participant warms up on the cycle ergometer for 5 minutes at low intensity (15 watts). Heart rate, blood oxygen saturation, and exertion (Borg scale) are then measured and recorded in the protocol.

**Main protocol**

Overall, the session will have a total duration of 60 minutes and will be divided into three parts: warm-up, main exercise period, and cool-down. The training will be performed on a cycle ergometer or treadmill (based on the patient's choice). The intensity will be based on maximum heart rate (HRmax), heart rate reserve (HRR), and the subjective perception of effort (Borg Scale), which will be monitored. The Borg scale will be the main variable considered to increase the protocol's intensity or stop the training, followed by the HRmax and the HRR, n this order. The HRmax will be calculated using a ramp protocol when the cycle ergometer is used or with Bruce’s protocol when a treadmill is used and confirmed by standard formulas.

**Borg scale**

The **Borg scale** will be the **main variable** considered to increase the exercise intensity or to stop the training. The Borg Scale aims to report the perception of effort during the exercise. The scale uses numerical values as descriptors to facilitate the understanding of the users (Pageaux, 2016).

The following sentences will be provided to the patients to explain the levels of the Borg scale: (a) 6 (“no exertion at all") – ‘this would be how you feel at rest’; (b) 7 - 9 (“very light exertion”) – like a slow walk; (c) 9 - 11 (“light”) – ‘you are comfortable and can continue for a long duration’; (d) 12 - 14 (“somewhat hard”) – you feel like you’re working, but can still continue without much difficulty; (e) 15 - 16 (“becomes hard”) – it’s challenging, but you can still continue; (f) 17 - 19 (“very hard”) – this is a difficult level of exertion, and you can do it but you really have to push yourself: (g) 20 (“maximal exertion”) – this is as hard as you can go and you likely cannot sustain this level for long.

The Borg Scale will be used during the evaluation of the modified Bruce´s Protocol for a treadmill or the ramp protocol for cycle ergometer and in the aerobic training to keep track of the effort and HR during the protocols.

**Heart rate submaximal (HRmax)**

The HRmax will be calculated through the modified Bruce´s Protocol for the treadmill training or by the ramp protocol for the cycle ergometer training.

**Modified Bruce’s protocol**

The **modified Bruce´s Protocol** is a treadmill test where the speed and the slope of the treadmill are increased systematically every three minutes. The Bruce submaximal protocol consisted of 3-min stages, where HR is recorded at the end of each stage. The first stage starts with 1.7 mph or 2.4 km/h (Metabolic equivalent of task (METs) = 5) without slope on the treadmill for three minutes. Then, the speed and the slope are increased each time for 3 minutes each interval until a maximum of 6.0 mph or 9.6 km/h with a slope of 22% (METs = 22) is achieved. The speed and the slope of the treadmill will be adjusted based on the participant’s fitness level, but participants are required to finish the three minutes at each level to progress to the next level. The test generally lasts between 6-15 min. The test will end when the participant reports exhaustion (“17” very hard” exercise), evaluated by the Borg scale (described above), and then the HR at this moment will be registered as the participant’s HR Bruce-max.

**Ramp protocol for the cycle ergometer**

Meanwhile, the **ramp protocol for the cycle ergometer** uses increments of the intensity of 5 to 50 w/minute to measure the HRmax, depending on the functional capacity of the participant. It is recommended that the increases be divided into equal values and applied at regular intervals of no more than 60 seconds. These increments could be determined for women by the formula proposed by Wasserman et al: [(height in cm – age) x 20] – [150 + (6 x weight in kg)] / 100. To be consistent with the values obtained by the modified Bruce´s Protocol and ramp protocol for the cycle ergometer, the Gellish equation to calculate the HRmax will be applied. This formula is: HRmax = 206.9 – (0.67 x age).

Both protocols will also be used to check the aerobic capacity of the participants after the treatment.

**Heart rate reserve (HRR)**

The heart rate reserve (HRR) will be calculated by the formula: HRR = HRmax – restHR. Heart rate at rest (restHR) will be measured after 30 minutes of lying position without any activity and sleep (the participant will be asked to arrive at least 30 minutes before the training). The restHR will be performed every week in the first session of the week to keep track of participant’s improvements and avoid underestimating the functional capability of the participant.

The control of the intensity is done based on the watts provided by the cycle ergometer. The watts are calculated based on the results from the aerobic capacity test (Ramp Protocol or Bruce’s protocol). We will consider the following references to calculate the watts for each level:

**Stage 1 (week 1-2):** HRmax = 60% (57 – 63%), HRR = 40% (30 - 39%), and level 9-11 on the Borg scale

**Stage 2 (week 2-6):** HRmax = 70% (64 - 76%), HRR = 40 – 59%, and level 12-14 on the Borg scale

**Stage 3 (week 6-12):** HRmax = 85% (77 - 95%), HRR = 60 – 89%, and level 15-17 on Borg scale

The intensity of the aerobic exercise program will be progressively increased according to each participant’s response but will be standardized as much as possible, as follows:

1) In the first two weeks, participants will start the walking/cycling program at low intensity (at 9 - 11 on the Borg scale, or < 40% (30 - 39%) HRR, or 60% (57 – 63%) HRmax);

2) 2 - 6 weeks: depending on the response to the first sessions, the training will progress to moderate intensity (12 - 14 on the Borg scale, or 40 - 59% HRR, or 70% (64 – 76)% HRmax);

3) In the last six weeks, High-Intensity Interval Training (HIIT) will be targeted as recommended by the literature. The HIIT consists of changing between moderate and high intensity for short intervals; the moderate intensity time is considered the active interval in this protocol. Thus, during the first four minutes, the participant should train at high intensity (15 - 17 on the Borg scale, or 60 - 89% HRR, or 75 - 90% HRmax), and in the subsequent three minutes, the participant should train at moderate intensity (12 - 14 on the Borg scale, or 40 - 59% HRR, or 70% (64 – 76)% HRmax). This is done continuously until the total training time is completed (4 min*4 times (high-intensity training) followed by 3 min of moderate training in between (active rest period), totaling 28 minutes of active training) according to the participant’s response.

The intensity of the aerobic training in each session will be targeted individually with an increase of five watts every five minutes according to the specific stage (when in low and moderate intensity levels) the participant is in and based on the Borg scale. The participant will progress to the next level if they accomplish the exercise intensity for the pre-established level without any discrepancy with the protocol. During protocols, the participants will wear the polar device to collect data related to HR and to ensure that the intensity of the training is being met.

It is important to gradually increase the exercise intensity according to the participant’s confidence and exercise tolerance because the individual can become more active, and goals should be set according to each participant's needs. It is expected that the intensity of the protocol does not exacerbate the participant’s pain perception, so the participant’s confidence and adherence to the exercises can be maintained. If the participant presents discomfort and/or an increase in pain perception during the exercise, the intensity will be regressed, or if the participant presents fatigue and asks to finish the protocol, the aerobic phase will be finished, and the cool-down phase will start. To control this, the Borg scale and the heart rate will be measured before starting the protocol, after the warm-up, during the aerobic exercise (every five minutes (or 4/3 minutes for the HIIT level)), immediately after the end of the protocol, and five minutes later.

**Cool down**

After completing the protocol, the participant rides the ergometer for 5 minutes at low intensity (15 watts), and blood oxygen saturation, pulse, and exertion are checked again. The legs are then stretched again (2x 30 seconds per side).

**REFERENCES**

Pageaux, B. (2016). Perception of effort in Exercise Science: Definition, measurement and perspectives. *European Journal of Sport Science*, *16*(8), 885–894. https://doi.org/10.1080/17461391.2016.1188992
