# Supplementary material for: Clinical significance of exercise‐induced hypoalgesia in individuals with temporomandibular disorders and neck pain: A clinical trial protocol
Source: Exp Physiol. 2025 Feb 24;111(6):2777–86. doi: 10.1113/EP091879 (PMC13238672; doi:10.1113/EP091879)
Supplement: Supplementary file 2 — Supplementary material 2. Description of the neck exercises for the neck training group. [file EPH-111-2777-s001.docx]

Supplementary Material 2 – Description of the neck exercises for the neck training group.

**Equipment needed:**

- NOD device (with pads if needed)
- Stabilizer Chattanooga
- Tablet and stand
- Elastic bands

| **Exercise** | **Guidance** | **Pictures** |
| --- | --- | --- |
| Neck mobility warm-up | Ask the participant to move the head and neck in different directions: **lateral inclination** (right and left), **flexion-extension** and **rotations** (right and left).  Always ask for pain or discomfort during these movements.  **2-3 times**, depending on tolerance of the participant. | **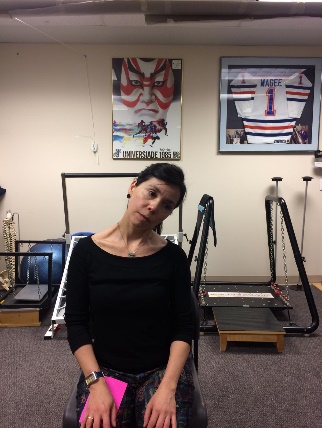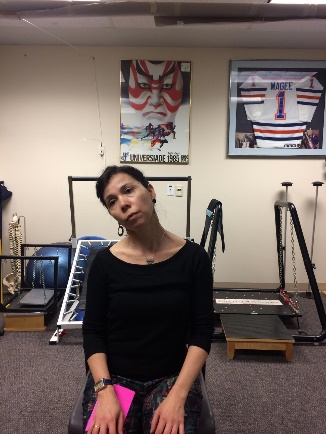**  **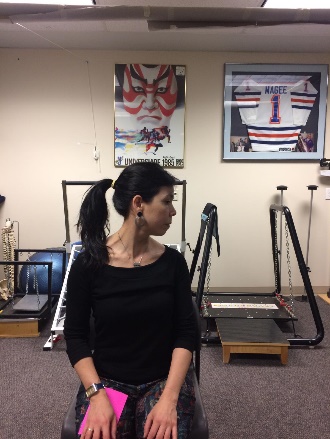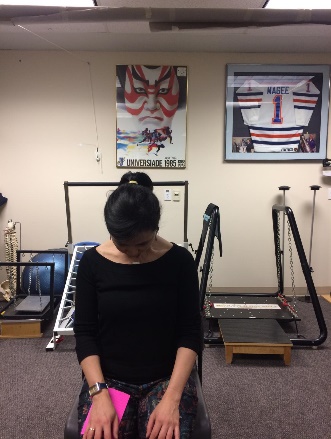**  **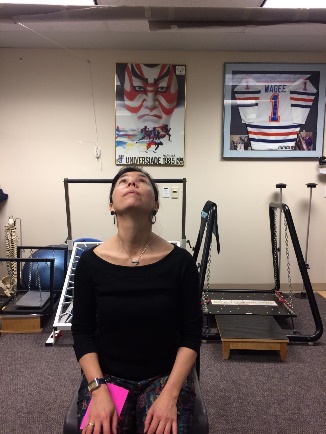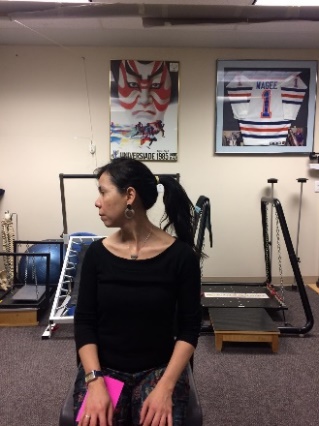** |
| Thoracic mobility | Ask the participant to imitate the movement of punching in an upper, horizontal and diagonal position, so the thoracic region moves during these movements.  The idea is that the participant generates the movement from the thoracic spine toward the upper extremities.  **10 times each side/20 in total in each position** (up, horizontal and diagonal). | **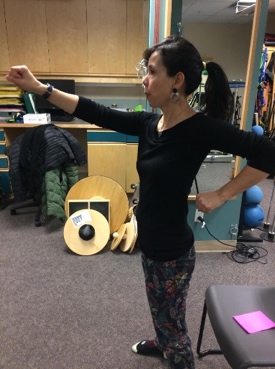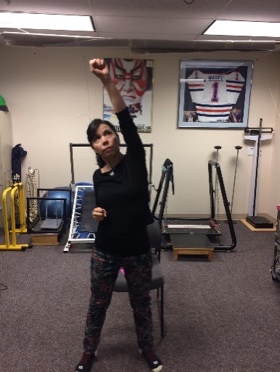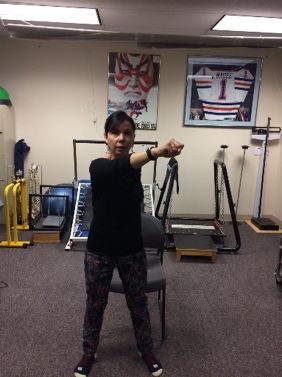** |
| Stretching | Stretching of the cervical and shoulder girdle muscles: upper trapezius, sternocleidomastoid, neck extensors, and pectoralis.  Each stretching position will be done passively by the therapist (first - third treatment) and then the participants will be instructed to do the stretching for each cervical muscle independently.  The participants could also be instructed to do the stretching using the contract-relax technique by telling them to contract against the hands of the therapist and then when relaxing they should push the head and neck in the direction of the stretch (gaining more amplitude).  Ideally the stretching should be maintained for **30 seconds for two times.** However, if the participants do not tolerate the hold, the therapist will start in the first sessions with 10 sec and progress until 30 sec are reached. | **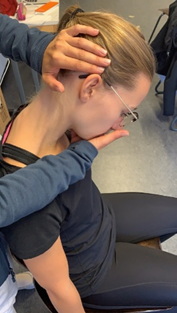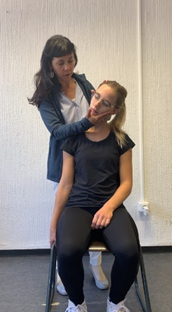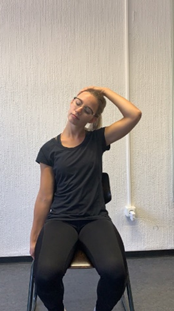**  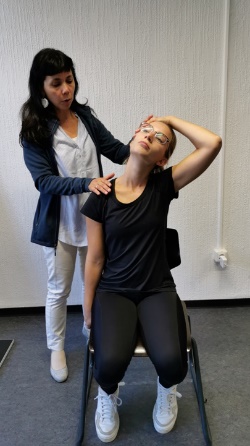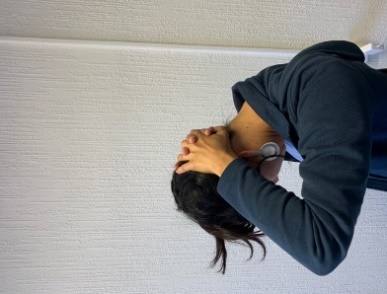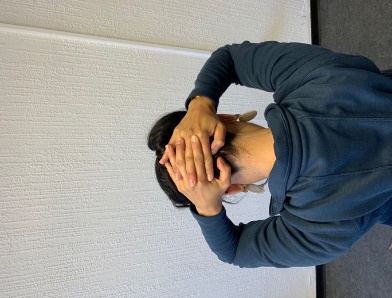 |
| Postural exercise | The postural exercise will be performed in sitting. The participant assumes an upright posture in a neutral lumbo-pelvic position and then gently lengthens the cervical spine by imagining they are lifting the base of their skull from the top of their neck. The participant is instructed to grow as much as possible, to lower the shoulders, bring the scapulas together and keep the spine in neutral position as much as possible.  During the treatment, the participants will be instructed to do this exercise **5 times**, holding the position for **10 seconds** each time. With the progress of the treatment the participants could improve the repetitions until **10 times**, based on their tolerance.  As home exercise, participants will be asked to perform the exercise, holding the position for 10 sec ideally every 15-20 min throughout their waking day. | **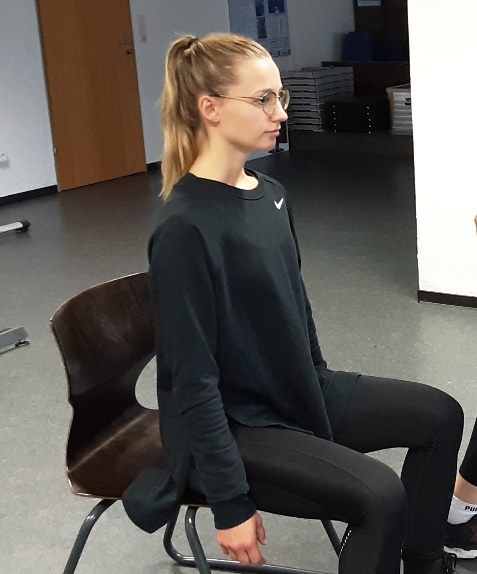** |
| Neck flexors training in upright or sitting position | Participants will be instructed to train their cervical flexor muscles in different positions. They progress the exercises to the sitting and upright positions.  The first part of the next stage consists of moving the head and cervical spine into an extension movement in the sitting or upright position. This phase of the movement involves an eccentric contraction of the cervical flexor muscles. Any compensation, such as chin retraction or cervical retraction, should be discouraged.  After this, participants are required to come back to neutral position of the cervical spine. This phase is done by contracting concentrically the cervical flexor muscles. It is important to check that the movement is initiated at the level of the craniocervical region rather than having a dominant action of the sternocleidomastoid.  The clinician can progress this exercise by increasing the range of the head extension movement as the control improves and second, by adding isometric hold exercises in different parts of the range of cervical returning movement (concentric flexion) to improve the cervical flexion synergy through functional ranges of extension.  The clinician must teach the participant to perform the movement correctly and to control and to eliminate any compensation strategy, such as neck retraction, excessive cervical flexion and/or jaw clenching. For the treatment to progress, the cranicervical flexion movement must be performed correctly. The wall and a mirror could be used a movement guidance when the participants are not familiarized with the movement.  The movement needs to be done **10 times.** However, the number of repetitions could be improved with time. At the first sessions the participants could do how many repetitions is needed and possible until she/he learns the movement correctly, without any discomfort. | 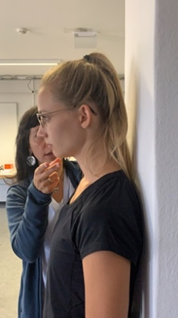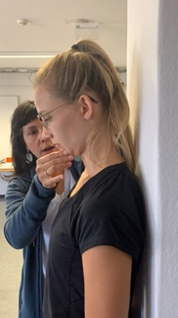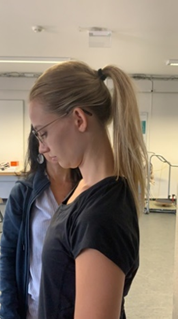  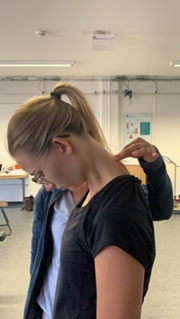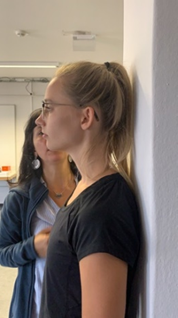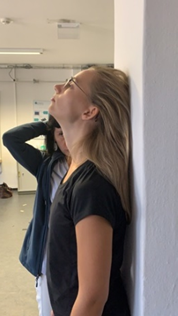 |
| Neck flexors training with the NOD device | Low-load craniocervical flexion exercises with the use of pressure biofeedback (NOD device).  Participants will be instructed to perform and hold progressively inner range positions of cranio-cervical flexion. During the task, the participants will be guided by feedback from a pressure unit placed behind the neck to monitor the slight flattening of the cervical lordosis, which occurs with the contraction of the longus colli.  Participants will be positioned in crook-lying position with the knees bended and pillows under the knees and asked to adjust the screen before training to ensure a comfortable position of the eyes.  Training of the cervical flexor muscles is done based on the evaluation of the craniocervical flexion test. Progression of this exercise will depend on the capacity of the participant to hold for at least **10 seconds** and perform **10 repetitions** in an appropriate way by level. They will use feedback from a pressure unit placed behind their neck at five pressure targets (from 20 mmHg to 30 mmHg). When this is done the exercise can be progressed.  High load cervical flexion exercises:  It will be implemented at the last 3-4 weeks of treatment (if not earlier), participants will be instructed to lift the head for 10 seconds and perform 10 repetitions.  If the participants cannot lift their head and maintain for 10 seconds, the cervical pressure device (Stabilizer) will be used to help with exercise.  This exercise will be only implemented when the participant can perform 10 repetitions lifting the head weight without pain (as described on “High load cervical flexion 10 RM evaluation and treatment guidance”). |  |
| High load cervical flexion 10 RM evaluation and treatment guidance | This evaluation may be conducted at the 7^th^ session (before or after) depending on the level of performance of the low load exercises with the feedback. The way the evaluation is performed is as follows:   - Participants performed a head lift exercise in the supine position. - The head lift exercise is taught, ensuring that the cranio-cervical spine is maintained in a neutral position while the head is lifted no further than 2 cm above the supporting surface for **2 seconds.** - During the evaluation session, each participant’s pain-free 10-repetition maximum (10RM) is assessed. - If the participant can perform 10 repetitions lifting the head weight and reported fatigue at the completion of the repetitions but not pain, then the exercise could be implemented during the training session. - If they are unable to perform 10 repetitions with head weight only, or if pain is present, the pressure biofeedback device is positioned under the occiput, inflated to 40 mm Hg and the participant is instructed to lift the head until the pressure reduced by 10 mm Hg (30mmHg). - If the participant is still unable to perform 10 repetitions in this manner, they are instructed to lessen the pressure change further (let’s say 35mmHg), until 10 repetitions could be performed. |  |
| Superficial neck flexors training | If 10 repetitions are performed easily with head weight, half-kilogram weight increments/ or an elastic band can be added to the forehead until the 10RM is found.  During the training session, participants perform **3 sets of 10 repetitions** at the predetermined intensity level. Each repetition could last approximately **3-5 seconds**, with rest intervals of 2 seconds between repetitions.  The holding time can also be increased, depending on the participant capacity. | **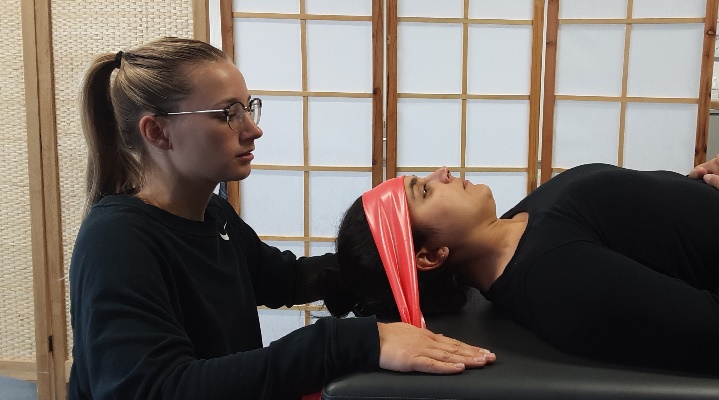** |
| Neck extensors training | The participant will be instructed to let the head and neck move into flexion, then to return to the starting position to train the eccentric/concentric function of the cervical extensors.  During the exercise, the participant is encouraged to maintain a neutral craniocervical position and instead, the flexion/extension motion is encouraged at the lower cervical spine. This can be facilitated by the therapist’s fingers.  To target the sub-occipital muscles, participants must be instructed to let the head and neck move into flexion and then to return to the starting position to train the eccentric/concentric function of the cervical extensor.  The clinician must teach the participant how to perform the movement. Attention should be given to the position of the head and neck and also to the scapula. Chin poking, for example, is one of the most common compensations seen with this movement and indicates excessive craniocervical extension, which usually is caused by dominance of the superficial muscles (e.g., semispinalis capitis).  Also, it is important to train the rectus capitis posterior major and minor muscles in recognition of their key proprioceptive function, their role in supporting and controlling the upper cervical joints and evidence of the changes that can occur in these muscles with neck involvement.  The participant must perform a cranio-cervical extension and flexion (head nodding) exercise whilst maintaining the mid and lower cervical spine in its neutral position.  Another exercise that can be done, facilitates the obliquus capitis superior and inferior and is a rotation movement of less than 40 degrees to focus rotation to the upper cervical region.  **10 repetitions** for each movement should be done.  The same type of exercises can be done in 4 points kneeling position.  The range of cervical extension through which the extensor muscles are trained should be progressively increased.  The number of repetitions and sets must also be increased as permitted by the participant’s response to the exercise and an endurance element could be incorporated by increasing the time the position is held, depending on the participants progress. | **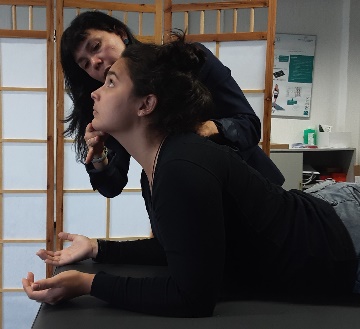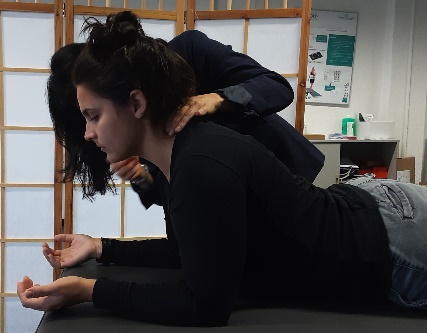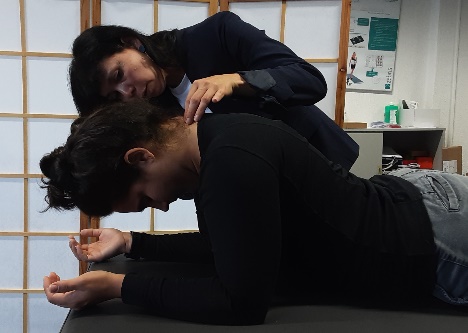** |
| Neck extensors exercise with elastic band | If the participant can control the movement of the head in flexion and extension in the prone position (elbow or kneeling position), then progression of the exercise could be performed by adding an elastic band as a resistance.  The participant is asked to contract/move the head against the elastic band and hold **3-5 seconds** in each position.  Depending on the performance of each participant, the participant could be asked to repeat this exercise **5, 10 or 15 times.** | 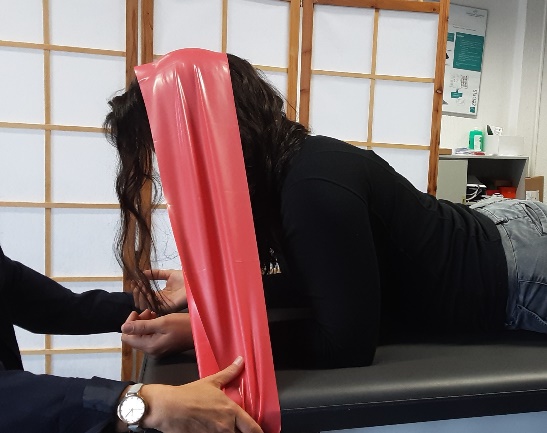 |

**Progression description from sessions 1 to 24:**

| **SESSION** | **1 visit** | **2 visit** | **3 visit** | **4 visit** | **5 visit** | **6 visit** | **7 visit** | **8 visit** | **9 visit** | **10 visit** | **11 visit** | **12- 24 visit** |  |
| --- | --- | --- | --- | --- | --- | --- | --- | --- | --- | --- | --- | --- | --- |
| **Neck mobility (flexion-extension, right and left lateral flexion, right and left rotation)** | | | | | | | | | | | | | |
| Repetitions | 3 / each | 3 | 3 | 3 | 3 | 3 | 3 | 3 | 3 | 3 | 3 | 3 |  |
| **Thoracic mobility (horizontal, cross and up)** | | | | | | | | | | | | | |
| Repetitions | 10 / each side | 10 | 10 | 10 | 10 | 10 | 10 | 10 | 10 | 10 | 10 | 10 |  |
| **Stretching (Upper trapez, SCM, extensor, pectoralis)** | | | | | | | | | | | | | |
| Repetitions | 2 | 2 | 2 | 2 | 2 | 2 | 2 | 2 | 2 | 2 | 2 | 2 |  |
| Holding time (sec) | 10 – 30 | 10 – 30 | 10 – 30 | 10 – 30 | 10 – 30 | 10 – 30 | 10 – 30 | 10 – 30 | 10 – 30 | 10 – 30 | 10 – 30 | 10 – 30 |  |
| **Postural exercise** | | | | | | | | | | | | | |
| Repetitions | 10 | 10 | 10 | 10 | 10 | 10 | 10 | 10 | 10 | 10 | 10 | 10 |  |
| Holding time (sec) | 5 | 5 | 5 | 5 | 10 | 10 | 10 | 10 | 15 | 15 | 15 | 15 |  |
| **Neck Flexor Training** | | | | | | | | | | | | | |
| **Neck flexor/extensor training in upright position (perform movement correctly)** | | | | | | | | | | | | | |
| Repetitions | 10 | 10 | 10 | 10 | 10 | 10 | 10 | 10 | 10 | 10 | 10 | 10 |  |
| Holding time  (sec inner range) | only correct performance | only correct performance | 5 | 5 | 5 | 10 | 10 | 10 | 10 | 15 | 15 | 15 |  |
| **Neck flexor training (feedback equipment - NOD) - low load** | | | | | | | | | | | | | |
| Pressure (%) | 20 | 20 | 40 | 40 | 60 | 60 | 80 | 80 | 100 | 100 | 100 | 100 |  |
| Repetitions | 10 | 10 | 10 | 10 | 10 | 10 | 10 | 10 | 10 | 10 | 10 | 10 |  |
| Holding Time (sec) | 5 | 10 | 5 | 10 | 5 | 10 | 5 | 10 | 5 | 10 | 10 | 10 |  |
| **Neck flexor training - high load**  **Session 1+2+3 without equipment: Flex and lift head** | | | | | | | | | | | | | |
| When the participant is able to do | **TEST RM max 10x 3sek ab ca. 4.-5. Session**  Training could be started here depending on the progression of the participant:  with the help of biofeedback (stabilizer) inflated to 40 mmHg **OR** without elastic band **OR** with elastic band | | | | | | | | | | | |  |
| Repetitions |  |  |  |  |  | 5 | 5 | 10 | 5 | 10 | 5 | 10 |  |
| Holding time (sec) |  |  |  |  |  | correct performance | correct performance | correct performance | 5 | 5 | 10 | 10 |  |
| **Different Positions: Flexion, Extension, Rotation** | | | | | | | | | | | | | |
| **Prone on elbow position OR on 4-kneeling position while maintaining the upper cervical spine in a NEUTRAL POSITION and lower cervical spine in FLEXION-EXTENSION**  This depends on each participant performance | | | | | | | | | | | | | |
| Repetitions | 10 | 10 | 10 | 10 | 10 | 10 | 10 | 10 | 10 | 10 | 10 | 10 |  |
| Holding time (sec) | only correct performance | only correct performance | 5 | 5 | 5 | 10 | 10 | 10 | 10 | 15 | 15 | 15 |  |
| **Prone on elbows position OR Prone on 4-kneeling position while maintaining the upper cervical spine in CERVICAL FLEXION and lower cervical spine in FLEXION-EXTENSION as well (all combined)** | | | | | | | | | | | | | |
| Repetitions | 10 | 10 | 10 | 10 | 10 | 10 | 10 | 10 | 10 | 10 | 10 | 10 |  |
| Holding time (sec) | only correct performance | only correct performance | 5 | 5 | 5 | 10 | 10 | 10 | 10 | 15 | 15 | 15 |  |
|  | | | | | | | | | | | | | |
| **Prone on elbows position OR Prone on 4-kneeling position while doing NECK ROTATIONS to both sides** | | | | | | | | | | | | | |
| Repetitions | 10 | 10 | 10 | 10 | 10 | 10 | 10 | 10 | 10 | 10 | 10 | 10 |  |
| Holding time (sec) | only correct performance | only correct performance | 5 | 5 | 5 | 10 | 10 | 10 | 10 | 15 | 15 | 15 |  |
| **Extensors with elastic bands** | | | | | | | | | | | | | |
| OBS: We should start when the participant is able to do it | | | | | | | | | | | | | |
| Repetitions |  |  |  |  |  |  |  |  | 10 | 10 | 5 | 10 |  |
| Holding time (sec) |  |  |  |  |  |  |  |  | 5 | 5 | 10 | 10 |  |
